# Cefepime MIC as a Predictor of the Extended-Spectrum β-Lactamase Type in *Klebsiella pneumoniae,* Taiwan

**DOI:** 10.3201/eid0805.010346

**Published:** 2002-05

**Authors:** Wen Leong Yu, Michael A. Pfaller, Patricia L. Winokur, Ronald N. Jones

**Affiliations:** * China Medical College Hospital, Taichung, Taiwan; † University of Iowa College of Medicine, Iowa City, Iowa, USA; ‡ JONES Group/JMI Laboratories, North Liberty, Iowa, USA; § Tufts University School of Medicine, Boston, Massachusetts, USA

**Keywords:** *Klebsiella pneumoniae*, extended-spectrum β-lactamases, CTX-M enzymes, carbapenems, cefepime, Taiwan

## Abstract

To guide selection of carbapenems or fourth-generation cephalosporins as therapy, 110 *Klebsiella pneumoniae* isolates with extended-spectrum β-lactamases from Taiwan were characterized by phenotypic (MICs), molecular, and chemical methods. MIC patterns of ceftazidime and cefepime clearly differentiate strains treatable by cefepime and those capable of efficiently hydrolyzing available cephalosporins (CTX-M series and SHV-types). Continued use of cefepime appears to be a treatment option in cases for which MIC results are available and interpreted by the criteria presented.

In recent years, extended-spectrum β-lactamase-producing *Klebsiella pneumoniae* (ESBL-KP) strains of the TEM, SHV, and CTX-M types have been discovered worldwide. Reference broth microdilution susceptibility rates (MIC <8 mg/L) for cefepime among ESBL-KP in various geographic regions show a wide range: Canada 94.4%, United States 87.6%, Western Pacific 76.1%, Europe 63.6%, and Latin America 49.6% (*1*). In Taiwan, the in vitro cefepime susceptibilities of ESBL-KP ranged from 37% to 100% (*2*,*3*). The gene encoding SHV-5 (pI 8.2) has been reported to be the most common ESBL in Klebsiellae in Taiwan (*2*,*4*). The CTX-M-3 (pI 8.4) enzyme has also been discovered in *Escherichia coli* isolates in southern Taiwan (*5*). For our study, we focused on the mechanisms of cefepime resistance among ESBL-KP isolates in Taiwan and attempted to predict cephalosporin therapeutic potentials by simple phenotypic patterns.

## The Study

We initially conducted reference broth microdilution tests (*6*,*7*) for 211 isolates of endemic and epidemic ESBL-KP from Taiwan; 53% of isolates had a cefepime MIC of <8 mg/L (susceptible). Isoelectric focusing (IEF) was then performed by the method of Matthew et al. (*8*). Approximately 40% of isolates had a pI of 8.2 enzyme (SHV-5); 40% of isolates produced enzymes with a pI of 7.9, 8.4, or 8.8 (CTX-M-type); an additional 20% of isolates contained both an SHV-5 plus a CTX-M enzyme.

The IEF results of 110 geographically representative isolates of ESBL-KP were categorized by cefepime MIC level (Table). The enzymes with pIs of 7.6 and 5.4 were SHV-1 and TEM-1, respectively, which have been reported previously in Taiwan hospitals (*2*,*4*). All the enzymes with pIs of 5.4, 7.6, and 8.2 were evenly distributed among the isolates regardless of cefepime MIC values, indicating no association with resistance to this fourth-generation cephalosporin. All 23 isolates with pI 8.2 enzymes and a nonsusceptible cefepime MIC (>16 mg/L) contained enzymes with pIs of 7.9, 8.4, or 8.8. In the absence of these CTX-M enzymes, isolates with pI 8.2 enzymes remained susceptible to cefepime. Thus, the high MIC level for cefepime was attributed to enzymes with pIs of 7.9, 8.4, and 8.8. This finding is supported by the fact that those isolates with a single CTX-M enzyme (10 with pI 7.9 enzymes [CTX-M-14] and 8 with pI 8.4 enzymes [CTX-M-3]) had very elevated cefepime MIC results in the absence of a pI 8.2 enzyme (*9*). Two isolates with pI 8.4 enzymes remained susceptible to cefepime (MIC 2 µg/mL) and probably produced low levels of CTX-M-3.

**Table:** Distribution of pI^a^ values in 110 isolates of extended-spectrum β-lactamase-producing *Klebsiella pneumoniae,* stratified by cefepime MIC level,^b^ Taiwan

Cefepime MIC (mg/L) (n=110)	pI 5.4 (n=89)	pI 6.3 (n=7)	pI 7.6 (n=92)	pI 7.8 (n=4)	pI 7.9 (n=40)	pI 8.2 (n=62)	pI 8.4 (n=43)	pI 8.8 (n=3)
>32 (n=38)	38	0	31	0	29^c^	20^d^	18^c^	2
16 (n=6)	6	0	5	0	5^c^	3^e^	5^d^	0
8 (n=26)	20	2^f^	22	0	6^g^	8^h^	12^g^	0
4 (n=19)	9	4^f^	17	1^f^	0	12^h^	6^g^	1^g^
2 (n=10)	7	1^f^	8	3^f^	0	8^h^	2^g^	0
<1 (n=11)	9	0	9	0	0	11^h^	0	0

^a^pI, Isoelectric point. Each strain may have multiple pIs. ^b^MIC test, reference broth microdilution method according to National Committee for Clinical Laboratory Standards. ^c^Ten of 34 isolates having a cefepime MIC >16 mg/L did not have enzymes with pI values of 8.2 or 8.4. ^d^ Eight of 23 isolates (cefepime MIC >16 mg/L) were not coexistent with pI 8.2 or 7.9. ^e^ All 23 isolates were simultaneously coexistent with pI 7.9, 8.4, or 8.8 (12 with pI 7.9 plus 8.4; 9 with pI 7.9; and 2 with pI 8.8). ^f^All the 11 isolates (pI 7.8 or 6.3) were coexistent with pI 8.2. ^g^Ceftazidime MIC <8 mg/L; ceftriaxone MIC <32 mg/L. ^h^Ceftazidime MIC <16 mg/L.

These data indicate that cefepime resistance in ESBL-KP isolates from Taiwan may result from either the cumulative effect of pI 7.9, 8.4, 8.8, or 8.2 enzymes or hyperproduction of any of the enzymes with the CTX-M phenotype (pI 7.9, 8.4, or 8.8). The enzyme with a pI of 8.8 is a novel CTX-M β-lactamase most similar to CTX-M-3 (unpub. data).

Several CTX-M enzymes have been shown to confer high MIC levels for cefepime (*10*–*12*). Bauernfeind et al. reported an isolate of *Salmonella typhimurium* that had a CTX-M-2 enzyme (pI 7.9) and a cefepime MIC of 64 mg/L (*10*). Outbreaks have also been reported of isolates producing CTX-M enzymes (pI 8.4), including *K. pneumoniae* (cefepime MIC 4-8 mg/L), *E. coli* (cefepime MIC 8-32 mg/L), and *Serratia marcescens* (cefepime MIC 16-64 mg/L) (*11*).

Szabo et al. reported an outbreak of 14 ESBL-KP strains (pI 8.2, probably SHV-5) that had high-level resistance to cefepime (MIC_90_ >256 mg/L) (*12*). Tzouvelekis et al. also noticed seven isolates of ESBL-KP (SHV-5) with cefepime MICs ranging from 32 mg/L to 64 mg/L. These researchers described the elevated cefepime MIC as being due to the combined effect of SHV-5 hyperproduction and decreased outer membrane permeability (loss of 36-kDa outer membrane protein [OMP]) (*13*). The cefepime MIC for isolates hyperproducing SHV-5 without loss of the 36-kDa OMP remained <16 mg/L, a susceptible level (*13*). Loss of the 36-kDa OMP also conferred cefoxitin resistance, and introduction of a plasmid carrying the 36-kDa OMP gene markedly reduced the MIC of cefoxitin, from 128 mg/L to 16 mg/L (*13*). Whether the isolates reported by Szabo et al. also had concomitant outer membrane defects is unknown, but these authors later recommended that cefepime not be considered the treatment of choice against SHV-5-producing ESBL-KP (*14*). Whether our cefepime-resistant isolates had a concomitant OMP defect is similarly unknown. However, in 44 isolates with cefepime MICs >16 mg/L, only 7 were resistant to cefoxitin. Furthermore, for isolates with high cefepime MIC values resulting from single CTX-M enzymes (10 with pI 7.9 and 8 with pIs of 8.4), only two (one each with pIs of 7.9 and 8.4) were resistant to cefoxitin. The relatively low rates of cefoxitin coresistance provide indirect evidence that 36-kDa OMP loss may not play an important role in the expression of cefepime resistance in ESBL-KP strains in Taiwan.

## Conclusions

Alternative therapy using cefepime against ESBL-KP strains in Taiwan could be reliable if appropriately guided by cefepime and ceftazidime MIC results. The cefepime MIC is useful for predicting the presence of CTX-M enzymes, which usually confer resistance to this fourth-generation cephalosporin. Cefepime cannot be used if the MIC exceeds 8 mg/L, which predicts the presence of CTX-M β-lactamases. Cefepime may reasonably be used clinically if the MIC is consistently <1 mg/L, which indicates the absence of a CTX-M enzyme. For isolates with cefepime MICs >2 to <8 mg/L, use of cefepime should be further guided by the ceftazidime MIC. If the ceftazidime MIC remains in the susceptible range (<8 mg/L, predicting enzymes of pI 7.9, 8.4, or 8.8), cefepime should not be used. If the ceftazidime MIC is >8 mg/L (predicting enzymes of pI 8.2), cefepime at appropriate doses has a potential therapeutic role because most pI 8.2 enzymes rarely elevate the cefepime MIC to >8 mg/L.

In conclusion, outer membrane defects and the inoculum effects (*13*) that may adversely elevate MIC values must still be considered if cefepime is chosen as an alternative therapy against ESBL-KP strains. This strategy of focused utilization of a newer cephalosporin could reduce some selective pressures of carbapenem use among ESBL-KP and thus minimize the development of carbapenem-resistant strains. In addition, phenotypic characteristics appear to accurately differentiate two important endemic and epidemic groups of ESBL types (CTX-M series and SHV-like) in *K. pneumoniae* strains in Taiwan.
